# Counteracting wetland overgrowth increases breeding and staging bird abundances

**DOI:** 10.1038/srep41391

**Published:** 2017-01-27

**Authors:** Petteri Lehikoinen, Aleksi Lehikoinen, Markku Mikkola-Roos, Kim Jaatinen

**Affiliations:** 1 The Helsinki Lab of Ornithology, Finnish Museum of Natural History, P.O. Box 17, 00014 Helsinki University, Finland; 2 Department of Biology, University of Lund, Sölvegatan 37, 223 62 Lund, Sweden; 3 Finnish Environment Institute, P.O. Box 140, 00251 Helsinki, Finland; 4 Tvärminne Zoological Station, University of Helsinki, J.A. Palménin tie 260, 10900 Hanko, Finland

## Abstract

Human actions have led to loss and degradation of wetlands, impairing their suitability as habitat especially for waterbirds. Such negative effects may be mitigated through habitat management. To date scientific evidence regarding the impacts of these actions remains scarce. We studied guild specific abundances of breeding and staging birds in response to habitat management on 15 Finnish wetlands. In this study management actions comprised several means of vegetation removal to thwart overgrowth. Management cost efficiency was assessed by examining the association between site-specific costs and bird abundances. Several bird guilds exhibited positive connections with both habitat management as well as with invested funds. Most importantly, however, red-listed species and species with special conservation concern as outlined by the EU showed positive correlations with management actions, underlining the conservation value of wetland management. The results suggest that grazing was especially efficient in restoring overgrown wetlands. As a whole this study makes it clear that wetland habitat management constitutes a feasible conservation tool. The marked association between invested funds and bird abundance may prove to be a valuable tool for decision makers when balancing costs and impact of conservation measures against one another.

Changes in land use, degradation of fresh water bodies and climate change are currently recognized to be among the main threats to global biodiversity^1^ the first two of which have direct effects on wetlands and their inhabitant organisms, while climate change affects both indirectly. Indeed it has been estimated that half of the world’s wetlands have been lost during the past century^2^, at the same time as many of the remaining ones have been degraded^3^. Draining and filling wetlands for agriculture and forestry are the main causes for global loss of wetlands while the major drivers of the ongoing degradation are alien species and eutrophication^4^. Despite their relatively small coverage of the world’s surface area, shallow waters comprise more than one third of global renewal ecosystem services^5^, including disturbance regulation, waste treatment, water and food supply, recreational and cultural values.

Waterbirds are a broad group of different taxa linked together by their preference for aquatic habitats. The ecosystem services provided by these birds include e.g. pest control, provisioning of food and materials as well as cultural services^6^. All of these ecosystem services are important to both westernized and indigenous communities, yet the total value of waterbirds to human kind has been overlooked until recent years^6^. Additionally, waterbirds have been shown to be valuable indicators of the state of wetlands^7^,^8^, mainly because they are readily able to move between wetlands and preferentially reside in the best available habitat^9^. However, large-scale destruction and degradation of wetlands may force migrating birds to stage at sub-optimal locations, thus impairing their chances of successful migration^10^. Habitat degradation may also have severe effects on waterbirds if the availability of suitable wetland habitat is insufficient to support the current populations. Anthropogenic eutrophication and water pollution have been raised as the most important problems facing the limnetic environment^11^. Although increased primary production in wetlands can be beneficial for waterbirds^12^, severe eutrophication and hypertrophy has been shown to impair waterbird numbers^7^,^8^. The negative effects of eutrophication are manifested through increased water turbidity^13^, increased cyprinid dominance in the fish fauna^14^ and overgrowth^15^,^16^. Increased turbidity decreases light penetration and causes diminished communities of submerged vegetation and invertebrates – both central food resources for waterbirds. Cyprinid dominated fish communities increase food competition and can lead to decreased food availability for waterbirds^14^. These fish often also disturb the bottom sediment while feeding and thereby increase turbidity and re-suspend nutrients, which in turn facilitates continued eutrophication^17^. Wetlands are especially vulnerable to plant invasions^18^. Such invasions can be initiated and speeded up by eutrophication^15^,^16^ and are usually detrimental to plant and animal diversity due to overgrowth and monocultures of one or very few dominant species^18^. In environments adversely affected by anthropogenic disturbance such invasions may be caused by both native and/or non-native plant species^19^. Reducing the external nutrient load is essential for solving problems regarding eutrophication, but due to the internal nutrient load, carried in the sediments of eutrophic wetlands, recovery may take decades^20^. These factors considered, eutrophication and overgrowth may need to be thwarted through wetland management by means of water regime manipulations and direct vegetation removal^21^ in order to restore habitat for waterbirds.

Given the importance of wetlands and the role of waterbirds as indicators of the state of wetlands, the scarcity of evidence-based knowledge regarding the effects of wetland management is notable^22^,^23^. This shortcoming is especially notable since great amounts of common funds are currently spent on wetland management projects for birds. Although highly valuable first steps have been taken in linking wetland management costs to waterbird abundance^24^, commonly applicable actions by which to prevent eutrophication-induced overgrowth remain unevaluated. While building artificial wetlands has been shown to increase biodiversity and reduce the amount of nutrients in eutrophicated wetlands^25^, little is known about the impacts of management actions on natural wetlands. A recent review on wetland management for birds^3^ called for the development of effective tools for predicting the effects of management for waterbirds on natural wetlands, and also suggested incorporating socioeconomic scenarios in the guidance of decision-making regarding wetland management. To heed this call we here set out to shed light on the ecological impacts of wetland management actions on several bird guilds, evaluate the cost efficiency of such management schemes and provide guidelines for future management of overgrown wetlands of natural origin as well as point out the areas of improvement.

To achieve these goals we assessed the effects of wetland management actions on waterbird abundances in Finland at 21 natural wetlands. The only prior habitat manipulation on these wetlands has been the small-scale use of shoreline meadows are pastures until 1950s, after which all grazing ceased on the sites. Altogether 15 of these wetlands were managed under the Life programme of the EU. All 21 studied wetlands are defined as SPAs (Special Protection Area for birds) in the EU Natura 2000 network of protected areas (Fig. 1). Based on the conservation values of Finnish SPA sites all the wetlands in this study are situated in the most valuable third of all the 450 SPA sites^26^. Moreover six of the wetlands are classified as Important Bird Areas (IBA) by BirdLife International and seven sites belong to the Ramsar wetland convention. Thus the study sites are the most valuable wetlands for birds in southern Finland, and almost half of them are important also on a national scale. The studied wetlands have been encumbered with nutrients since the 1950s^27^. These nutrients derived from both unpurified wastewaters from human settlements (ceased in the 1970s) and from fertilizer run-off from agriculture (still ongoing)^13^,^28^. This has resulted in the ecological state of the wetlands, as outlined by the EU Water Framework Directive (2000/60/EC), deteriorating from good to poor; with an exception of two sites currently classified as moderate. The original habitat of the study sites has been open meadow consisting of common reed (*Phragmites australis*) free low-sward vegetation and smaller patches of the high-sward plant species water horsetail (*Equisetum fluviatile*) and lakeshore bulrush (*Schoenoplectus lacustris*). In both natural succession processes and in invasive processes the common reed has been shown to establish homogenous high-sward populations, especially in response to environmental disturbance such as eutrophication^18^,^29^. This will eventually cause the gradual filling of shallow wetlands and a corollary reduction in biodiversity^18^,^30^. This process was observed on all the study sites, starting in the 1960s and resulting in severe overgrowth. Thick stands of common reed had overtaken the whole shoreline as well as the meadows, thereby reducing either the coverage of shrubs or the extent of open water, or both. Eutrophication and overgrowth are seen as major causes of population declines in many waterbird species, leading them to be declared of national red-list status^31^. Management actions in this study were aimed to restore open coastal meadows and areas of open water by eradicating parts of the common reed monocultures. The purpose of these actions was to increase habitat diversity and provide necessary meadow habitat for the use of waders, dabbling ducks, geese and several passerines^32^,^33^ as well as open water areas for birds feeding by diving^34^. The aim was however also to retain stands of common reed for birds favouring high-sward vegetation, such as reed bed passerines, the bittern and rallids^35^,^36^,^37^ (Fig. 2). Management actions included mechanic cutting and harrowing of vegetation on land, cattle grazing, tree and shrub removal and removing aquatic vegetation and bottom sediment by dredging (Fig. 2). The effect of these actions was observed by counting birds on each wetland before the management started, and again after three and nine years of management. To study potentially different responses to management by birds differing in ecology and/or behaviour we identified ten bird guilds based on food type, foraging behaviour and breeding habitat preferences: (i) dabbling ducks, (ii) diving omnivores, (iii) diving piscivores, (iv) swans, (v) geese, (vi) waders, (vii) black-headed gull, (viii) rallids and bittern, (ix) open habitat passerines and (x) shrub and reed bed passerines (for guild specific species see Supplementary Table S1). We hypothesized that management would increase the number of individuals in general, but we were also interested in the guild-specific associations to several management actions. We also assessed the cost efficiency of wetland management by testing the correlation between the total costs of wetland specific management and total waterbird abundances. In order to assess the effects of wetland management on species most in need of conservation we conducted separate analyses for EU red-listed species^38^ (hereafter red-listed species) and EU Birds Directive Annex I species (hereafter Annex I species) (for group specific species see Supplementary Table S1) elucidating the effects of management actions on bird abundance. To the best of our knowledge this is the first study where wetland management actions are linked to bird abundances on a wide scale by studying the impacts of several different management methods on several ecological guilds across three seasons, while also considering the cost-efficiency of these management actions.

## Results

In spring the number of staging migrants correlated positively with grazing area (F_1,323_ = 47.28, *P* < 0.0001) and was also affected by area of cutting and harrowing (guild × cutting/harrowing interaction: F_7,323_ = 4.60, *P* = 0.0001; for a visual example of effect sizes see Fig. 3; for full information on guild specific effect sizes see Supplementary Table S4). This interaction showed that the numbers of dabbling ducks and waders were positively correlated with the extent of cutting and harrowing (Supplementary Table S4). Our data also showed a significant correlation between number of staging birds and extent of dredging, but also this effect differed between guilds (guild × dredging interaction: F_7,323_ = 3.00, *P* = 0.005). This interaction showed that the number of diving piscivores was negatively correlated with the extent of dredging (Supplementary Table S4), whereas the number of waders and black-headed gulls were positively correlated with the extent of dredging (Supplementary Table S4).

The number of breeding birds was positively affected by grazing area (F_1,11_ = 18.60, *P* = 0.001) and was also affected by dredging area, but this effect differed between guilds (guild × dredging interaction: F_10,113_ = 2.39, *P* = 0.01). This interaction showed that the numbers of breeding diving piscivores was negatively correlated with the dredged area (Supplementary Table S5), whereas the numbers of breeding black-headed gulls and rallids and bittern were positively correlated with dredging area (Supplementary Table S5).

In autumn the number of staging migrants was affected by grazing area, but this effect differed between guilds (guild × grazing area interaction: F_7,310_ = 8.45, *P* < 0.0001; for a visual example of effect sizes see Fig. 4; for full information on guild specific effect sizes see Supplementary Table S6). This interaction showed that the number of dabbling ducks, geese, waders and black-headed gulls were positively correlated with grazing area (Supplementary Table S6).

The number of staging spring migrants was affected by the total costs of wetland management, but these effects differed between guilds (guild × total cost interaction: F_7,332_ = 8.76, *P* < 0.0001). This interaction showed that the number of dabbling ducks, geese, waders and black-headed gulls were positively correlated with the total sum of funds invested in wetland management (Supplementary Table S7).

The number of breeding birds was affected by the total costs of wetland management, but these effects differed between guilds (guild × total cost interaction: F_10,113_ = 4.77, *P* < 0.0001; for a visual example of effect sizes see Fig. 5; for full information on guild specific effect sizes see Supplementary Table S8). This interaction showed that the number of diving piscivores was negatively correlated with the invested funds, whereas the numbers of black-headed gulls, rallids and bittern and open habitat passerines were positively correlated with the total sum of funds invested in wetland management (Supplementary Table S8).

The number of staging autumn migrants was affected by the total costs of wetland management, but these effects differed between guilds (guild × total cost interaction: F_7,310_ = 9.14, *P* < 0.0001). This interaction showed that the number of dabbling ducks, geese, waders and black-headed gulls were positively correlated with the total sum of funds invested in wetland management (Supplementary Table S9).

In all six ecological and total cost models, the number of birds prior to management period and size of water area were positively connected with observed bird abundance (Supplementary Tables S4–S9). In contrast, the size of land area of the wetland did not correlate with bird numbers in any of the models (Supplementary Tables S4–S9).

The number of red-listed staging spring migrants was positively correlated with grazing area (b = 0.06, F_1,31_ = 14.84, *P* < 0.001; for full model see Supplementary Table S10). The number of Annex I species staging during spring migration was positively correlated with grazing area (b = 0.03, F_1,31_ = 6.16, *P* = 0.02; for full model see Supplementary Table S11).

The number of red-listed staging autumn migrants was positively correlated with areas of grazing (b = 0.03, F_1,26_ = 9.48, *P* = 0.029), cutting and harrowing (b = 0.03, F_1,26_ = 6.25, *P* = 0.041) and dredging (b = 0.04, F_1,26_ = 4.32, *P* = 0.047) (Supplementary Table S12). We found no significant correlations between any management action and the number Annex I species staging during autumn migration.

The number of red-listed staging spring migrants showed a trend indicating a positive correlation with the total cost of wetland management (b = 0.000009, F_1,31_ = 3.28, *P* = 0.08). We found no significant correlation between the cost of wetland management and the number of Annex I species staging during spring migration (b = 0.000004, F_1,31_ = 0.78, *P* = 0.38).

The number of red-listed staging autumn migrants was positively correlated with the total cost of wetland management (b = 0.000008, F_1,28_ = 5.16, *P* = 0.034; for full model see Supplementary Table S13). We found no significant correlation between the cost of wetland management and the number of Annex I species staging during autumn migration (b = 0.00001, F_1,28_ = 1.38, *P* = 0.18).

## Discussion

The results show that management was correlated with a rapid increase in waterbird numbers on the studied wetlands. The most salient finding of this study was that bird abundances in every guild showed a positive correlation with one or more management actions and all actions except tree and shrub removal exhibited a positive correlation with the bird numbers of at least one guild. Cattle grazing together with cutting and harrowing were found to be solely positively correlated with the number of birds on wetlands. Against our predictions grazing and cutting did not show a negative correlation with the number of breeding shrub and reed bed passerines despite their breeding habitat being largely reduced by these actions. This can possibly be a result of increased food abundance in the cut reed bed^35^ and due to sufficient breeding habitat being spared despite the grazing and cutting of vegetation. We did, however, detect a negative effect of dredging on the number of birds in the diving piscivore guild.

In addition to pointing out potentially central actions for wetland management against overgrowth, the results show that the costs of management were in general positively correlated with the number of birds frequenting the wetlands. The number of breeding diving piscivores was the sole exception to this finding as it showed a negative correlation with the amount of invested funds. From the perspective of the birds in most dire need of conservation grazing was the single most efficient management action. During spring migration both red-listed and Annex I species showed a positive correlation with this management action, as did and red-listed species during the autumn migration. The numbers of red-listed species staging on the wetlands during autumn also increased after dredging and after cutting and harrowing. While the exact mechanisms by which each management action impacts a given guild remains unknown, and beyond the scope of the current study, it is unlikely that the observed effects are mere artefacts of annual fluctuations in bird abundances since the bird numbers prior to management were taken into account in the analyses and the bird counts were done during the exact same years on both managed and unmanaged sites.

Our results underline the central role of livestock grazing in the preservation of ecological diversity in open habitats^39^ as grazing was found out to be the single management action with positive connection to the most diverse group of birds. Grazing creates a mosaic of vegetation of different length and structure, which provides shelter^39^, increases the biodiversity of grassland plants and invertebrates^40^,^41^ and thereby also favours many bird species^42^. Indeed our findings are supported by recent studies on wetlands suggesting that a complex habitat with diverse vegetation height increases avian species richness^43^, and reducing common reed by grazing and burning increases the number of various breeding birds^23^. The presence of cattle and its dung has been shown to increase and diversify the insect community, thus providing ample food for insectivorous birds^41^,^44^. Cattle presence has also been shown to increase the reproductive success of a passerine bird and the quality of offspring produced^44^. These ecological effects of grazing are likely to be at least partly responsible for our findings that show a positive correlation between grazing and the abundance of a broad range of avian taxa. Furthermore, a positive response to grazing increases the number of individual birds, such as dabbling ducks and waders, staging on the grazed pasture, which in turn may facilitate a positive response in other guilds as the safety in numbers increases^45^. In such cases the likelihood of detecting a predator increases^46^, while the likelihood of any individual of falling victim to predation is reduced^47^. Despite the host of positive effects brought about by grazing in wetland habitats, it is worth noting that cattle may trample bird nests on the ground^48^, wherefore it is important to keep the grazing pressure low or moderate during the breeding season^49^ or bring cattle to the pasture after wader chicks and ducklings have hatched.

Together with grazing, cutting and harrowing was the other action showing only positive correlations and it benefitted dabbling ducks and waders on spring migration and increased the numbers of endangered species in autumn. The removal of emergent hydrophytes by mowing has been shown to increase invertebrate densities and, as a corollary, dabbling duck abundances^50^. It is possible that this is also the mechanism yielding the observed positive effect of cutting and harrowing on dabbling duck abundances. Besides the diversifying effect of livestock grazing both grazing and mechanic cutting increases the amount of open area in the landscape, which can in itself be largely beneficial for several bird groups. Waders thrive in short vegetation where locomotion and feeding is easier^32^ and where approaching predators can be more readily detected^51^,^52^. These benefits are likely also available for waterfowl since wintering ducks have been shown to be more abundant on lakes without onshore reed beds^7^. Prior to management actions the shorelines and coastal meadows surrounding most of the wetlands in this study were overwhelmed by common reed, the removal of which resulted in improved the feeding opportunities of waterfowl, but more markedly in the feeding opportunities of waders changing from almost non-existent to abundant.

Dredging was positively associated with the number of waders and black-headed gulls in spring, with the numbers of breeding black-headed gulls, rallids and bitterns, and with the abundance of red-listed staging birds during autumn migration. Dredging found out to be negatively correlated with the number of diving piscivores both during the spring and summer. This effect may be explained by the increased turbidity dredging causes in the water column. Diving piscivores are mainly visual predators and rely strongly on their vision during pursuit of prey fish. Increased water turbidity may impair their feeding efficiency^53^, resulting in piscivores preferring areas with clearer water. The numbers of rallids and bitterns, on the other hand, exhibited a positive correlation to the extent of dredging. The main reason for the positive correlation is likely to be the increased amount of preferred habitat. Rallids and especially bitterns prefer fringes and tracts in wet emergent vegetation and avoid even-aged drier and denser vegetation^36^,^37^,^54^. The availability of such habitat increases with the extent of dredging that creates a mosaic of channels in the emergent vegetation.

Despite the general positive associations between bird abundances and wetland management shown by the results, swans and diving omnivores and piscivores were an exception by exhibiting clearly fewer positive associations to management than the other guilds. The reasons for this remain unclear, however the populations of these groups of birds have recently undergone relatively strong changes, where swans have increased and many diving omnivores and piscivores have declined^31^. While our analyses do take into account population sizes prior to management actions, the population changes these groups have undergone may indeed be strong enough to mask potential effects of habitat management. A more likely, however not mutually exclusive, explanation for the modest impact of management on both swans and diving omnivores and piscivores may be that the main body of management work carried out was aimed at improving the habitat on land and far less effort was put on improving the underwater habitats.

Total cost of management was positively correlated with bird numbers in many guilds during different seasons, suggesting that invested funds facilitate habitat improvement and the increase of waterbirds. The number of breeding diving piscivores exhibited a negative correlation with the total funds invested in management, which is likely explained by the fact that dredging is relatively expensive and may hamper piscivores’ feeding by increasing water turbidity. From a conservation point-of-view as well as from a socioeconomic point-of-view it is reassuring that the amount of funds invested in wetland management was not only positively correlated with the abundance of common species, but also with that of red-listed species.

As a result of national agricultural policy farms raising cattle are not as common in southern Finland as they are in central and northern parts of the country^55^. As a result the cattle grazing the study wetlands were rented by environmental administration authorities and transported to the wetlands, both of which increased the costs arising from grazing. In areas where cattle farms are more abundant and in closer proximity of managed wetlands both farmers and wetland conservation may achieve significant benefits. In such cases farmers could under controlled terms use the wetlands as pastures for their cattle and costs arising from maintaining fences could be shared.

Taken together our results show that wetland management can mitigate the negative effects of eutrophication-induced overgrowth by common reed on a host of waterbirds throughout their life histories. Eutrophication-induced overgrowth and biodiversity loss due to plant invasions are worldwide problems^11^,^15^, wherefore these findings may be widely applicable where large monoculture-like high-sward plat populations encroach on native habitats. We believe that this is especially true in North America where the common reed overgrows an array of aquatic habitats, diminishing bird populations^56^ and reducing biodiversity on a large scale^18^. Moreover, the results are important as several waterbird species have already declined and become red-listed in Europe^38^, most probably due to ongoing eutrophication and overgrowth of open areas^31^,^57^. Effective management actions to mitigate overgrowth are urgently needed as a climate change induced increases in precipitation are suggested to increase nutrient flow into boreal wetlands in Europe^58^ thus exacerbating current eutrophication and overgrowth related problems. At the same time, climate change is likely to increase the importance of these wetlands for waterfowl as staging and wintering areas since many waterfowl species have markedly delayed their autumn migration^59^, and even begun to winter at higher latitudes than previously^60^,^61^. These changes mean that waterfowl are likely to spend an increasing proportion of their annual cycle on boreal wetlands. Because of this we suggest – in addition to establishing and/or extending protected area networks^61^,^62^ – the implementation of habitat management schemes on wetlands of high importance for waterbirds. Such schemes would maintain these wetlands as high quality habitat for these animals and allow for the long term study of the effects of habitat management actions (see also ref. 63).

## Conclusions

Our results show that the breeding and staging habitats of waterbirds can be enhanced by wetland management in general, and by grazing in particular, as it was shown to be the single most efficient management action. This study gives a scientific foundation for the notion that habitat management can indeed increase waterbird numbers, which have great value for both society and conservation^6^, at the same time as enhancing the ecosystem services provided by wetlands. These habitat improvements are especially striking since the studied wetlands were the most valuable and internationally important wetlands for birds in southern Finland, and thus high-quality habitat already prior to management. The fact that such high-quality habitat may be further enhanced for a host of bird guilds underlines the improvement potential well-targeted management may offer wetlands currently in poorer state than the ones in our current study.

## Methods

### Site and study setup

This study was conducted on 21 eutrophic wetlands in southern Finland during the years 2003–2012. Characteristic for the sites is that they all are of natural origin and quite shallow in depth and freeze on winter when hardly any birds are present or any primary production occurs. The wetlands are non-tidal fresh or brackish water inland lakes or bays of the Baltic Sea, respectively. Management was conducted during two periods: first in years 2004–2006 and second in 2007–2012. Birds on all wetlands were counted in the autumn of 2003 and in the spring of 2004 before management started. Birds were again counted after the periods of management (autumns 2006 and 2012 and springs 2007 and 2012) to evaluate the effects of management on waterbirds. These periods differ from one another in respect to management schemes since all sites were initially unmanaged. At 13 sites management actions were continued in the second period.

Each managed wetland was divided into managed and unmanaged sections. As the management actions were small-scaled the area within a radius of 500 m from the actual management was considered as a managed section. If two managed areas were situated more than 1 km from each other they were considered as separate managed sections. Area outside the radius of 500 m were considered as an unmanaged section. If an unmanaged section of water on a single wetland was split by land it was considered as two separate sections. These classifications resulted in a total of 35 wetland sections out of which management was performed at 17 sections in the first, and at 13 sections in the second, management period (Supplementary Tables S2 and S3).

### Management actions

**Figure 2:**
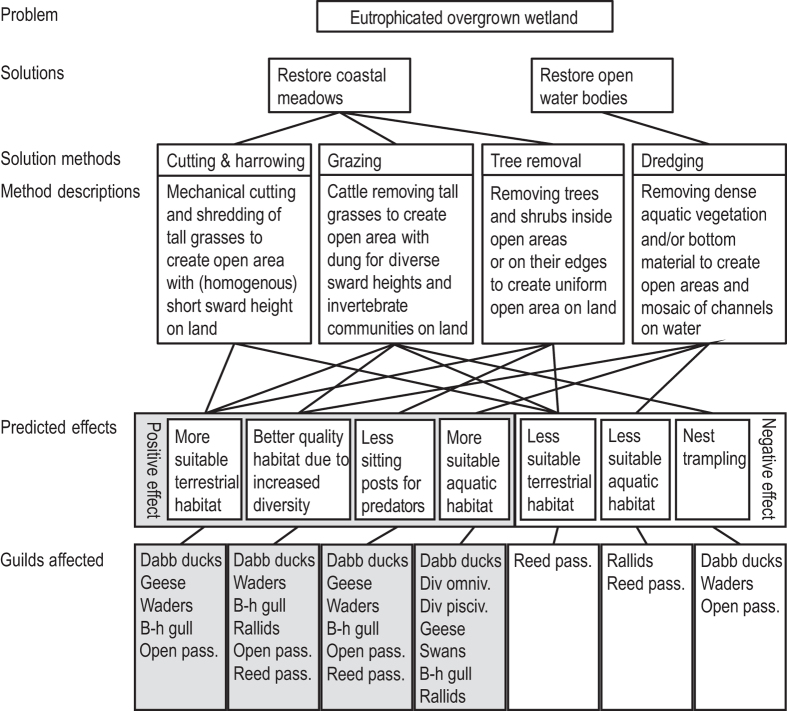
Diagram of the management rationale, aims and predicted effects on the studied bird guilds. Abbreviations: Dabb duck = dabbling ducks, Div omniv. = diving omnivores, Div pisciv. = diving piscivores, B-h gull = black-headed gull, Rallids = rallids and bittern, Open pass = open habitat passerines, Reed pass. = shrub and reed bed passerines.

**Figure 3:**
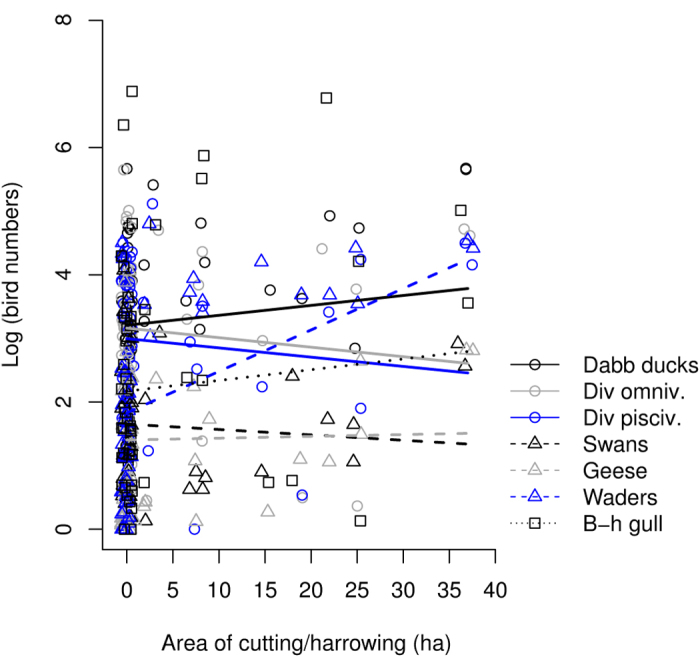
The guild specific responses of staging spring migrant birds to the extent of the cut and harrowed area on the wetlands. Dabbling ducks and waders exhibited statistically significant positive associations with cutting and harrowing.

**Figure 4:**
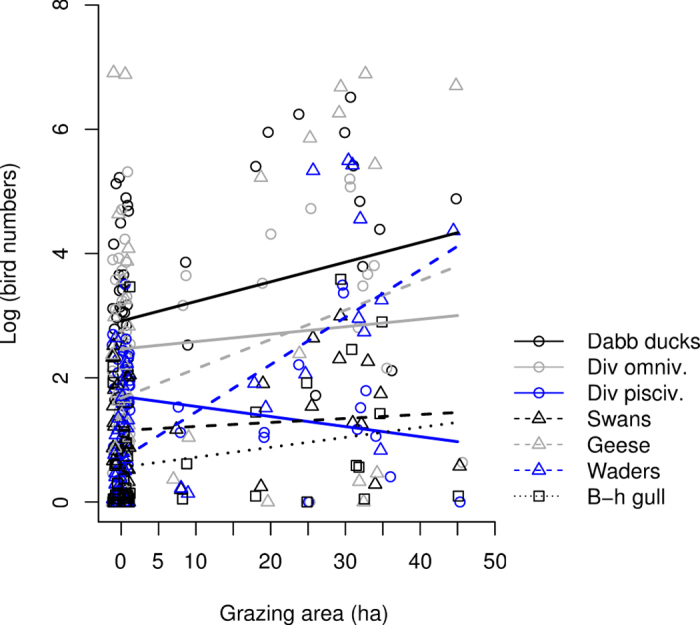
The guild specific responses of staging spring migrant birds to the extent of grazed area. Dabbling ducks, geese and waders exhibited statistically significant positive associations with grazed area on the wetlands.

**Figure 5:**
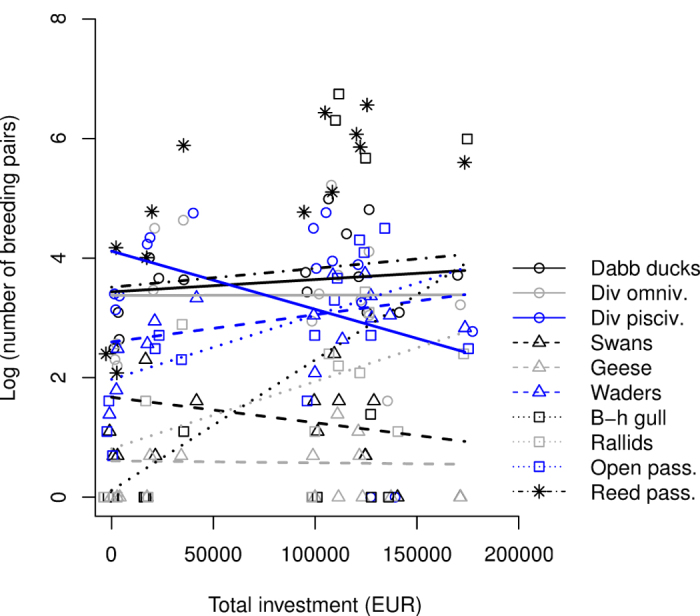
The guild specific responses of breeding birds to the total funds invested in management. The amount of breeding pairs of black-headed gull, rallids and bittern and open area passerines showed significant positive associations with the amount of invested funds. Diving piscivores exhibited a significant negative association with the amount of invested funds.

The primary aims of the management were reducing eutrophication driven overgrowth caused mainly by common reed, and restoring coastal meadows and open bodies of water (Fig. 2). The actions entailed commonly used methods for restoring open habitats^21^: i) mechanical cutting and harrowing of reed beds, ii) cattle grazing on pastures edging the wetlands, iii) tree and shrub removal (hereafter: tree removal) and iv) dredging of both impenetrable submerged and emergent aquatic vegetation and bottom sediment (Fig. 2).

Coastal meadows were restored by cutting and harrowing on 11 sites in the first, and 7 in the second, management period. The mean areas ( ± SD) of cutting and harrowing were 16.6 ± 12.4 ha (15% of the land area of these sites) in the first and 7.7 ± 3.6 ha (10%) in the second period. Seven sites were grazed by cattle in both periods and the mean grazed areas were 25.8 ± 8.8 ha (19.4%) and 27.7 ± 11 ha (20%) in the first and second periods, respectively. The mean grazing pressure was moderate and constituted of 0.96 ± 0.31 animals ha^−1^ in the first period and 0.81 ± 0.26 cows ha^−1^ in the second period. Dredging of aquatic vegetation was done at 10 sites in the first and 4 in the second period and the total areas dredged were 96.27 ± 8.6 ha (7.2% of the water area of these sites) in the first and 21 ± 2.3 ha (10%) in the second period. Tree removal was done in 12 sites in the first and 2 sites in the second period with total areas of 72.43 ± 3.2 ha (6% of the land areas of these sites) and 4.3 ± 2.6 ha (2%) in the first and second periods, respectively. The site-specific volumes and costs of management are presented in Supplementary Tables S2 and S3. Tree removal occurred on managed areas partly in overlap with grazing and cutting and harrowing. Since the main purpose of tree removal is to exclude sitting posts for predators on otherwise open areas^64^ the overlap between management actions was considered to be unimportant. Because of this we considered the effects of each management action independently in the analyses.

The total costs of management actions in this study were 1.75 M€, with 1.08 M€ and 0.68 M€ were spent on management actions during the first and second periods, respectively. The salaries of administrative or bird counting personnel were not included nor taken into account. Costs associated with the separate actions were as follows: grazing: 375,000 € and 568,500 €, dredging: 373,000 € and 19,000 €, cutting: 252,000 € and 87,000€, and tree removal: 80,000 € and 5,500 € in the first and second period of management, respectively.

### Bird data

The numbers of staging birds were counted by experienced birdwatchers throughout the spring migration seasons (beginning of April to end of May) in years 2004, 2007 and 2012. In the spring of 2007, an additional count was conducted at the end of March on study sites 1–8 (see Supporting Tables S2 and S3) because of early ice breakage following an exceptionally warm and early spring. Autumn migration counts (mid-July to mid-November) were conducted in years 2003, 2006 and 2012. During the survey periods, counts were performed approximately once every five days according to a set schedule of dates. In total 15 and 20 counts were conducted in spring and autumn, respectively. Counts were conducted from set observation points from which the entire section of the wetland being counted, either managed or unmanaged, was visible. The breeding birds were censused in April–June 2004 and 2007 either by counting pairs from the set observation points at the beginning and at the end of May (guilds 1–5 & 7; Supporting Table S1) or by mapping all bird territories by walking through the whole wetland once every two weeks (guilds 6 and 8–10, Supporting Table S1). An active territory was counted as one breeding pair, which is the standard unit of breeding bird censuses in Finland. The numbers of birds were separately counted on the managed and the unmanaged sections of the study sites. As the management was done during summer and the counts took place during the following autumn and spring the effect of the management should have been roughly equivalent in both seasons because of the lack of plant and algal growth over winter. An exception was made in year 2012 when the post-management counts were conducted first in spring; the management data from the previous summer was nonetheless used to explain bird numbers (see statistical analyses below) as was done for the previous years.

We summed species-specific bird numbers for each season into ten separate guilds based on food type, foraging behaviour and breeding habitat preferences (Supplementary Table S1). The guilds of rallids and bittern, open habitat passerines and shrub and reed bed passerines were only included in the breeding season counts because they are difficult to detect with the methods used to survey staging birds. Classifying the black-headed gull (*Larus ridibundus*) as a guild of its own is justified because of its key role it plays in providing shelter against predators for wetland birds during the breeding season^65^. Diving waterbirds were split into omnivores and piscivores based on their feeding habits, since piscivores are more dependent on sight when catching prey^53^ and therefore more vulnerable to changes in water quality and turbidity. All recorded species, guilds and numbers of individuals are shown in Supplementary Table S1.

The autumn migration periods of dabbling ducks and diving omnivores, diving piscivores, swans and geese start in late July or early August in southern Finland^66^. Therefore, the counts for these guilds in the first two autumn counts (approx. 15–22 July) were excluded because individuals observed during this time are likely not migrants, but rather breeding birds. For the same reason, waterfowl broods and the numbers of black-headed gulls observed during the last two spring counts (approx. 21–30 May) were not included in to the spring totals.

Some of the prescheduled bird counts could not be conducted due to bad weather or lack of resources. If a count was performed but interrupted by bad weather (e.g. thick fog and/or heavy rain) it was discarded. In total 46 (2.6%) out of 1752 counts were missing. To minimize the effect of discarded counts during migration season, seasonal sums were divided by the number of counts. A site was omitted from further analyses if more than 20% of counts were missing. This resulted in the exclusion of one study site out of 21 in each management period (Supplementary Tables S2 and S3).

Breeding bird numbers were counted on the entire site without the division into managed and unmanaged sections. This was done to see how the management affected the whole population of a breeding guild as the management was assumed to harm some of them by diminishing their breeding habitat^67^. The seasonal sums of breeding birds were counted as breeding pairs instead of individuals, as in spring and autumn migration sums. In two cases we combined two nearby sites to achieve sufficient sample size allowing analysis (sites 2–3 and 13–18; Supplementary Tables S2 and S3), because they were sections of the same large lake. Due to a lost notebook, breeding data of the shrub and reed bed passerine guild was missing at two sites, and these sites were excluded from further analyses considering these guilds.

### Statistical analyses

In order to study the responses of wetland inhabiting birds to varying levels of wetland management we created a linear mixed model (LMM) for each season (spring migration, breeding and autumn migration) in which the number of birds at each wetland section was explained by the extent (area) of cutting and harrowing, cattle grazing, tree removal and dredging (Supplementary Tables S2 and S3). Thus, the response variable comprises two observations from each site in each management period: one observation prior to management and one after management actions were implemented. According to this logic, on areas that were not managed all observations of management actions equalled zero. The response variable was log-transformed to accommodate potential non-linear increases in the number of birds on the wetlands. To account for the lack of independence and potential pseudo replication arising repeated observations, wetland identity was set as a random factor in the analysis. To study bird guild specific responses to the management actions we included bird guild and the two-way interaction between bird guild and each management action. Because of their main effect being the removal of annual plants, which can re-grow rapidly after discontinued management, only the previous summer’s effect of cutting, harrowing and cattle grazing were considered. On the other hand the summed extent of all prior tree removal and dredging was considered because of the long-lasting effects of these management actions. Because cutting and harrowing both represent mechanical removal of annual vegetation we combined these two management actions to form a single variable in the analyses. In addition to the management actions the land and water areas of each site, as well as the number of birds in the previous count were included in the models to account for varying sizes of wetlands and for varying avian population sizes prior to the management actions.

In order to elucidate how the total costs of wetland management are associated with total bird abundances we constructed a LMM for each season where the number of birds was explained by the total cost of the management actions, bird guild, land area, water area and the number of birds prior to each management period. To study bird guild specific responses to the costs of management we included bird guild and the two-way interaction between bird guild and total cost in the model.

In order to assess how management actions affect red-listed species and Annex I species we constructed a LMM for both red-listed and Annex I species for the spring and autumn migration periods, where the number of these threatened birds were explained by the extent of cutting and harrowing, cattle grazing, tree removal and dredging, as well as by land area, water area and the number of birds prior to management period. Finally, we studied how the total costs of wetland management are associated with the numbers of red-listed species and Annex I species, by constructing a LMM for both red-listed and Annex I species for the spring and autumn migration periods, where the number of these threatened birds were explained by the total cost of the management actions, land area, water area and the number of birds prior to management period. In all four model options mentioned above the identity of the wetland was included as a random effect to account for repeated observations from the same wetlands. Data from the breeding season was not sufficient for management action and cost analyses in red-listed and Annex I species.

All statistical analyses were performed using the software R 3.1.2. The residuals of all models adhered to the assumption of normality. Models were reduced by stepwise removal of non-significant variables, whereby only significant variables and two-way interactions were retained in final models (Supplementary Tables S4–S13). However, the population size prior to management period, land area and water area were retained in all models, regardless of significance, to correct for differences in bird populations and wetland size. For all significant two-way interactions the guild-specific slopes were tested against the null-hypothesis that the slope is equal to zero. The non-significant variables left outside the final models are presented in Supplementary Tables S14–S20.

### Data Availability

Data deposited in the Finnish Biodiversity Information Facility/FinBIF. http://tun.fi/HR.1925.

## Additional Information

**How to cite this article**: Lehikoinen, P. *et al*. Counteracting wetland overgrowth increases breeding and staging bird abundances. *Sci. Rep.* **7**, 41391; doi: 10.1038/srep41391 (2017).

**Publisher's note:** Springer Nature remains neutral with regard to jurisdictional claims in published maps and institutional affiliations.

## Supplementary Material

Supplementary Tables

## Figures and Tables

**Figure 1:**
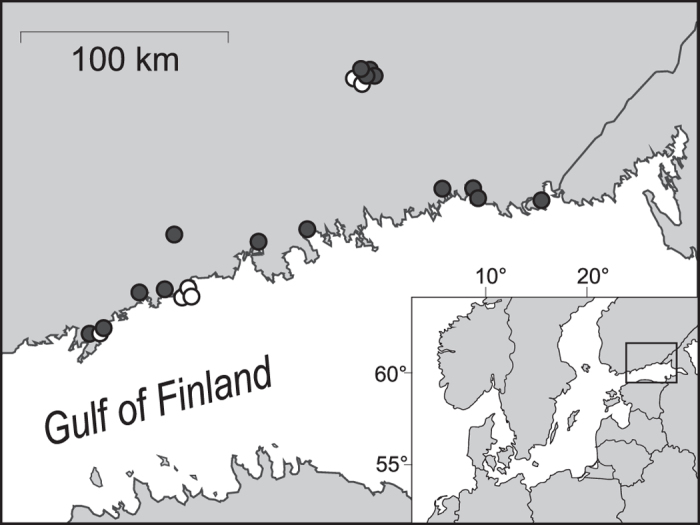
Localities of the wetlands studied. Dark circles represent the 15 managed wetlands and white circles the 6 unmanaged wetlands under study. These two maps were generated using the software MapInfo Professional (version 12.5, http://www.mapinfo.com/product/mapinfo-professional) and combined using the software CorelDRAW (version Graphics Suite X7, http://www.coreldraw.com/en/product/small-business-software/).
