# Supplementary material for: Counteracting wetland overgrowth increases breeding and staging bird abundances
Source: Sci Rep. 2017 Jan 27;7:41391. doi: 10.1038/srep41391 (PMC5269664; doi:10.1038/srep41391)
Supplement: Supplementary Tables [file srep41391-s1.pdf]

Counteracting wetland overgrowth increases breeding and staging bird abundances

Petteri Lehikoinen[1, 2*], Aleksi Lehikoinen[1], Markku Mikkola-Roos[3], Kim Jaatinen[4]

[1] *The Helsinki Lab of Ornithology, Finnish Museum of Natural History, P.O. Box 17, 00014 Helsinki University, Finland;* [2] *Department of Biology, University of Lund, Sölvegatan 37, 223 62 Lund, Sweden;*[3] *Finnish Environment Institute, P.O. Box 140, 00251 Helsinki, Finland,* [4] *Tvärminne Zoological Station, University of Helsinki, J.A. Palménin tie 260, 10900 Hanko, Finland.*

* Correspondence author. E-mail: Petteri.Lehikoinen@helsinki.fi, telephone +358407233383.

Supplementary information

Supplementary Table S1. Compositions of guilds and total species specific sums of birds counted in different seasons before and after the two management periods. In guilds 8–10 spring and autumn numbers, an abbreviation “na” stands for “not available” since the numbers of these guilds were not counted during migration seasons. Species belonging to the EU red-list[26] are marked with asterisk (*) and the EU Birds Directive Annex I (Council Directive 2009/147/EC) with circumflex accent (^). Naming follows The Howard & Moore Complete Checklist of the Birds of the World.

Guild	Species	Scientific name	n		
			spring	autumn	breeding
1 Dabbling ducks	Eurasian Wigeon*	*Mareca penelope**	30189	15336	174
	Gadwall	*M. strepera*	216	1633	3
	Common Teal	*Anas crecca*	26229	22609	166
	Mallard	*A. platyrhychos*	36012	46526	950
	Northern Pintail*	*A. acuta**	2095	374	3
	Garganey*	*Spatula querquedula**	391	60	33
	Northern Shoveler	*S. clypeata*	3192	4350	111
	Common Shelduck	*Tadorna tadorna*	13	7	1
2 Diving omnivores	Long-Tailed Duck*	*Clangula hyemalis**	9	223	0
	Common Eider*	*Somateria mollissima**	58	0	0
	Velvet Scoter*	*Melanitta fusca**	3	7	0
	Common Scoter	*M. nigra*	4	207	0
	Common Goldeneye	*Bucephala clangula*	14841	5449	342
	Common Pochard*	*Aythya ferina**	6545	2434	99
	Ferruginous Pochard^	*A. nyroca^*	1	0	0
	Tufted Duck	*A. fuligula*	25489	11055	140
	Greater Scaup*	*A. marila**	36	229	0
	Little Grebe	*Tachybaptus ruficollis*	2	4	0
	Slavonian Grebe*^	*Podiceps auritus*^*	36	19	1
	Common Coot	*Fulica atra*	16168	25080	614
3 Diving piscivores	Smew^	*Mergellus albellus^*	3021	615	0
	Goosander	*Mergus merganser*	22529	1832	78
	Red-breasted Merganser*	*M. serrator**	205	13	12
	Red-throated Diver^	*Gavia stellata^*	26	7	0
	Black-throated Diver^	*G. arctica^*	150	49	0
	Red-Necked Grebe	*Podiceps grisegena*	537	144	45
	Great Crested Grebe	*P. cristatus*	23841	7639	1198
	Great Cormorant	*Phalacrocorax carbo*	1307	480	0
4 Swans	Mute Swan	*Cygnus olor*	4477	3582	74
	Tundra Swan*^	*C. columbianus bewickii*^*	615	20	0

		Whooper Swan^	*C. cygnus*^	6687	789	10
5	Geese	Brent Goose	*Branta bernicla*	0	5	0
		Barnacle Goose^	*B. leucopsis*^	1591	98157	0
		Red-breasted Goose^	*B. ruficollis*^	0	2	0
		Canada Goose	*B. canadensis*	2713	5925	9
		Snow Goose	*Anser caerulescens*	0	3	0
		Pink-footed Goose	*A. brachyrhynchos*	1	1	0
		Greylag Goose	*A. anser*	1777	11200	2
		Bean Goose	*A. fabalis*	677	1153	0
		Greater White-fronted Goose	*A. a. albifrons*	31	1635	0
6	Waders	Eurasian Oystercatcher*	*Haematopus ostralegus**	567	62	0
		Grey Plover	*Pluvialis squaterola*	12	8	0
		Eurasian Golden Plover^	*P. apricaria*^	13	82	0
		Common Ringed Plover	*Charadrius hiaticula*	173	247	0
		Little Ringed Plover	*C. dubius*	312	112	10
		Northern Lapwing*	*Vanellus vanellus**	4601	11301	70
		Whimbrel	*Numenius phaeopus*	30	0	0
		Eurasian Curlew*	*N. arquata**	855	66	18
		Bar-tailed Godwit^	*Limosa lapponica*^	0	26	0
		Black-tailed Godwit*	*L. limosa**	9	1	0
		Ruddy Turnstone*	*Arenaria interpres**	0	1	0
		Red Knot	*Calidris canutus*	0	1	0
		Ruff*^	*C. pugnax**^	1846	2278	0
		Broad-billed Sandpiper	*C. falcinellus*	27	5	0
		Curlew Sandpiper*	*C. ferruginea**	0	40	0
		Temminck's Stint	*C. temminckii*	183	53	0
		Dunlin	*C. a. alpina*	3	462	0
		Little Stint	*C. minuta*	2	60	0
		Buff-breasted Sandpiper	*C. subruficollis*	0	1	0
		Eurasian Woodcock	*Scolopax rusticola*	3	1	0
		Great Snipe*^	*Gallinago media**^	0	2	0
		Common Snipe	*G. gallinago*	2693	2647	197
		Jack Snipe	*Lymnocryptes minimus*	83	61	0
		Common Sandpiper	*Actitis hypoleuca*	874	450	95
		Green Sandpiper	*Tringa ochropus*	355	125	14
		Spotted Redshank	*T. erythropus*	426	122	0
		Common Greenshank	*T. nebularia*	1205	359	0
		Common Redshank*	*T. totanus**	1069	55	76
		Wood Sandpiper^	*T. glareola*^	5899	3014	0
		Marsh Sandpiper*	*T. stagnatilis**	1	0	0
		Red-necked Phalarope^	*Phalaropus lobatus*^	9	0	0
7	Black-headed gull	Black-headed Gull	*Chroicocephalus ridibundus*	68689	4218	2684
8	Rallids & bittern	Eurasian Bittern^	*Botaurus stellaris*^	na	na	39
		Western Water Rail	*Rallus aquaticus*	na	na	87
		Corncrake^	*Crex crex*^	na	na	8
		Spotted Crake^	*Porzana porzana*^	na	na	30

		Little Crake^	*Zapornia parva*^	na	na	2
		Common Moorhen	*Gallinula chloropus*	na	na	14
9	Open habitat	Meadow Pipit*	*Anthus pratensis**	na	na	188
	passerines	Yellow Wagtail	*Motacilla flava*	na	na	202
		Citrine Wagtail	*M. citreola*	na	na	8
		White Wagtail	*M. alba*	na	na	122
		Eurasian Sky Lark	*Alauda arvensis*	na	na	33
		Whinchat	*Saxicola rubetra*	na	na	100
		Northern Wheatear	*Oenanthe oenanthe*	na	na	4
		Red-backed Shrike^	*Lanius collurio*^	na	na	27
		Bearded Reedling	*Panurus biarmicus*	na	na	48
10	Shrub and reed bed	Common Rosefinch*	*Erythrina erythrina**	na	na	224
	passerines	Eurasian Reed Bunting	*Schoeniclus schoeniclus*	na	na	1404
		Savi's Warbler	*Locustella luscinioides*	na	na	5
		River Warbler*	*L. fluviatilis**	na	na	8
		Grasshopper Warbler	*L. naevia*	na	na	12
		Sedge Warbler	*Acrocephalus schoenobaenus*	na	na	3502
		Blyth's Reed Warbler	*A. dumetorum*	na	na	16
		Marsh Warbler	*A. palustris*	na	na	44
		Common Reed Warbler	*A. scirpaceus*	na	na	627
		Great Reed Warbler	*A. arundinaceus*	na	na	98
		Common Whitethroat	*Curruca communis*	na	na	193
		Thrush Nightingale	*Luscinia luscinia*	na	na	183

Supplementary Table S2. The site-specific extent and costs of management actions on first managing period 2004–2007. Wetlands belonging to Ramsar convention are marked with asterisk (*) and to IBA (Important Bird Areas assigned by BirdLife International) with circumflex accent (^).

	Wetland	Section	Land area	Water area	Volume (ha)						Total costs (euros)				
					Cutting & harrowing	Cattle grazing	Cattle (amount)	Grazing pressure (cows/ha)	Tree removal	Dredging	Cutting & harrowing	Cattle grazing	Tree removal	Dredging	Total
1	Saltfjärden	1	129.4	5.6	25	25	30	1.2	6.8	0	29500	90000	11000	7000	137500
	Saltfjärden	2	50	5	0	0	0	0	0	0	0	0	0	0	0
2	Norra Fladet	3	44	21	25	32	20	0.625	10.4	0	33000	62000	3000	0	98000
	Norra Fladet	4	0	90	0	0	0	0	0	0	0	0	0	0	0
3	Morsfjärden	5	13	119	0	0	0	0	0	0	0	0	0	0	0
4	Laajalahti*^	6	70.5	102.5	36.5	30	20	0.66667	0	0	52000	59500	0	0	111500
	Laajalahti*^	7	0	75	0	0	0	0	0	0	0	0	0	0	0
5	Tuusulanjärvi	8	30	55	0	0	0	0	4	14	0	0	1500	26000	27500
	Tuusulanjärvi	9	20	135	0	0	0	0	2	5	0	0	4000	41000	45000
	Tuusulanjärvi	10	27	63	0	0	0	0	8	4	0	0	11000	90000	101000
6	Vanhankaupunginlahti*^	11	193	92	37	32	50	1.5625	0.8	0.27	24500	54000	2500	44000	125000
	Vanhankaupunginlahti*^	12	133	206	0	0	0	0	0	0	0	0	0	0	0
7	Porvoonjoen suisto*^	13	75	48	21.5	19	15	0.78947	0	10	20000	16000	0	37000	73000
	Porvoonjoen suisto*^	14	15	70	0	8	8	1	0	0	0	27000	0	0	27000
	Porvoonjoen suisto*^	15	105	390	0	0	0	0	6.7	0	0	0	8000	0	8000
8	Pernajanlahti*	16	104.8	50.2	8.6	35	30	0.85714	6	10	34000	65000	5000	20000	124000
	Pernajanlahti*	17	15	70	0	0	0	0	0	0	0	0	0	0	0
9	Salminlahti	18	31	151	9.6	0	0	0	7	0	11800	0	8640	0	20440
10	Kirkkojärvi*^	19	50	72	7.7	0	0	0	0	33	23600	0	0	39600	63200
	Kirkkojärvi*^	20	96	15	0	0	0	0	12.5	5	0	0	15374	45360	60734
11	Pappilansaari-Lupinlahti*^	21	20	129	0	0	0	0	0	0	0	0	0	0	0
	Pappilansaari-Lupinlahti*^	22	15	117	0	0	0	0	0	0	0	0	0	0	0
	Pappilansaari-Lupinlahti*^	23	6	107	4.6	0	0	0	5.2	0	11800	0	6396	0	18196
12	Kirkon-Vilkkiläntura*^	24	19	71	na	na	na	na	na	na	na	na	na	na	na
	Kirkon-Vilkkiläntura*^	25	5	25	na	na	na	na	na	na	na	na	na	na	na
	Kirkon-Vilkkiläntura*^	26	73	15	na	na	na	na	na	na	na	na	na	na	na
13	Jaalanlahti	27	46	39	7	0	0	0	3	9	11300	0	3700	13500	28500
14	Kyrönlahti	28	20	20	0.3	0	0	0	0	6	500	0	0	9000	9500
15	Lintukymi	29	13	55	0	0	0	0	0	0	0	0	0	0	0
16	Mukulanlahti	30	27	98	0	0	0	0	0	0	0	0	0	0	0
17	Suolalahti	31	15	100	0	0	0	0	0	0	0	0	0	0	0
18	Tervolanlahti	32	22	71	0	0	0	0	0	0	0	0	0	0	0
19	Bruksviken	33	23	18	0	0	0	0	0	0	0	0	0	0	0
20	Porvarinlahti	34	50	30	0	0	0	0	0	0	0	0	0	0	0
21	Torpviken	35	16.8	13.8	0	0	0	0	0	0	0	0	0	0	0

Supplementary Table S3. The site-specific extent and costs of management actions on second managing period 2008–2012. Wetlands belonging to Ramsar convention are marked with asterisk (*) and to IBA (Important Bird Areas assigned by BirdLife International) with circumflex accent (^).

	Wetland	Section	Land area	Water area	Volume (ha)						Total costs (euros)				
					Cutting & harrowing	Cattle grazing	Cattle (amount)	Grazing pressure (cows/ha)	Tree removal	Dredging	Cutting & harrowing	Cattle grazing	Tree removal	Dredging	Total
1	Saltfjärden	1	129.4	5.6	0	45	35	0.77778	0	0	0	168000	0	0	168000
	Saltfjärden	2	50	5	0	0	0	0	0	0	0	0	0	0	0
2	Norra Fladet	3	44	21	5	32	20	0.625	0	0	4000	87000	0	0	91000
	Norra Fladet	4	0	90	0	0	0	0	0	0	0	0	0	0	0
3	Morsfjärden	5	13	119	0	0	0	0	0	0	0	0	0	0	0
4	Laajalahti*^	6	70.5	102.5	3	30	20	0.66667	0	0	8000	72500	0	0	80500
	Laajalahti*^	7	0	75	0	0	0	0	0	0	0	0	0	0	0
5	Tuusulanjärvi	8	30	55	0	0	0	0	0	0	0	0	0	0	0
	Tuusulanjärvi	9	20	135	0	0	0	0	0	0	0	0	0	0	0
	Tuusulanjärvi	10	27	63	0	0	0	0	0.3	0	0	0	2000	0	2000
6	Vanhankaupunginlahti*^	11	193	92	8	24.7	32	1.29555	0	0	5000	67500	0	0	72500
	Vanhankaupunginlahti*^	12	133	206	0	0	0	0	0	0	0	0	0	0	0
7	Porvoonjoen suisto*^	13	75	48	5	19	8	0.42105	0	0	10000	40000	0	0	50000
	Porvoonjoen suisto*^	14	15	70	0	8	8	1	0	0	0	35000	0	0	35000
	Porvoonjoen suisto*^	15	105	390	0	0	0	0	0	0	0	0	0	0	0
8	Pernajanlahti*	16	104.8	50.2	8	35	30	0.85714	4	0	25000	89000	3500	0	117500
	Pernajanlahti*	17	15	70	0	0	0	0	0	0	0	0	0	0	0
9	Salminlahti	18	31	151	0	0	0	0	0	0	0	0	0	0	0
10	Kirkkojärvi*^	19	na	na	na	na	na	na	na	na	na	na	na	na	na
	Kirkkojärvi*^	20	na	na	na	na	na	na	na	na	na	na	na	na	na
11	Pappilansaari-Lupinlahti*^	21	20	129	0	0	0	0	0	0	0	0	0	0	0
	Pappilansaari-Lupinlahti*^	22	15	117	0	0	0	0	0	0	0	0	0	0	0
	Pappilansaari-Lupinlahti*^	23	6	107	0	0	0	0	0	0	0	0	0	0	0
12	Kirkon-Vilkkiläntura*^	24	19	71	0	0	0	0	0	0	0	0	0	0	0
	Kirkon-Vilkkiläntura*^	25	5	25	0	0	0	0	0	0	0	0	0	0	0
	Kirkon-Vilkkiläntura*^	26	73	15	6	0	0	0	0	0	14391	6058	0	0	20449
13	Jaalanlahti	27	46	39	15	0	0	0	0	3	20250	0	0	3240	23490
14	Kyrönlahti	28	20	20	0	0	0	0	0	3	0	0	0	2835	2835
15	Lintukymi	29	13	55	0	0	0	0	0	7.5	0	0	0	6750	6750
16	Mukulanlahti	30	27	98	0	0	0	0	0	0	0	0	0	0	0
17	Suolalahti	31	15	100	0	0	0	0	0	7.5	0	0	0	6075	6075
18	Tervolanlahti	32	22	71	0	0	0	0	0	0	0	0	0	0	0
19	Bruksviken	33	23	18	0	0	0	0	0	0	0	0	0	0	0
20	Porvarinlahti	34	50	30	0	0	0	0	0	0	0	0	0	0	0
21	Torpviken	35	16.8	13.8	0	0	0	0	0	0	0	0	0	0	0

Supplementary Table S4. Summary table of the management actions in the final LMM explaining the abundance of staging migrant birds on the studied wetlands during their spring migration.

Independent variable	Parameter estimate (b)	df	t	P
**Birds prior to management**	**0.005**	**323**	**7.84**	**<.0001**
**Water area**	**0.009**	**323**	**4.78**	**<.0001**
Land area	-0.004	323	-1.45	0.15
Guild diving omnivores	0.005	323	0.21	0.83
Guild diving piscivores	-0.02	323	-0.09	0.93
**Guild swans**	**-1.5**	**323**	**-6.95**	**<.0001**
**Guild geese**	**-1.77**	**323**	**-8.18**	**<.0001**
**Guild waders**	**-1.47**	**323**	**-6.83**	**<.0001**
**Guild b-h gull**	**-1.08**	**323**	**-5.06**	**<.0001**
**Grazing**	**0.05**	**323**	**6.56**	**<.0001**
**Guild dabbling ducks × Cutting/harrowing**	**0.02**	**323**	**1.02**	**<.0001**
Guild diving omnivores × Cutting/harrowing	-0.01	323	-0.98	0.33
Guild diving piscivores × Cutting/harrowing	-0.01	323	-0.95	0.34
Guild swans × Cutting/harrowing	-0.008	323	-0.55	0.58
Guild geese × Cutting/harrowing	0.003	323	0.18	0.85
**Guild waders × Cutting/harrowing**	**0.07**	**323**	**4.25**	**<.0001**
Guild b-h gull × Cutting/harrowing	0.02	323	1.10	0.27
Guild dabbling ducks × Dredging	0.03	323	1.19	0.24
Guild diving omnivores × Dredging	-0.01	323	-0.50	0.62
**Guild diving piscivores × Dredging**	**-0.06**	**323**	**-2.36**	**0.02**
Guild swans × Dredging	0.005	323	0.20	0.84
Guild geese × Dredging	0.02	323	0.60	0.55
**Guild waders × Dredging**	**0.08**	**323**	**2.97**	**0.003**
**Guild b-h gull × Dredging**	**0.05**	**323**	**1.98**	**0.048**

Supplementary Table S5. Summary table of the management actions in the final LMM explaining the abundance of breeding birds on the studied wetlands.

Independent variable	Parameter estimate (b)	df	t	P
**Birds prior to management**	**0.006**	**113**	**4.92**	**<.0001**
**Water area**	**0.003**	**11**	**4.25**	**0.001**
Land area	-0.001	11	-0.74	0.47
Guild diving omnivores	-0.27	113	-0.63	0.53
Guild diving piscivores	0.14	113	0.33	0.74
**Guild swans**	**-2.13**	**113**	**-5.01**	**<.0001**
**Guild geese**	**-3.09**	**113**	**-7.24**	**<.0001**
Guild waders	-0.77	113	-1.82	0.07
**Guild b-h gull**	**-2.15**	**113**	**-5.09**	**<.0001**
**Guild rallids and bittern**	**-2.39**	**113**	**-5.63**	**<.0001**
**Guild open hab. passerines**	**-0.84**	**113**	**-1.99**	**0.049**
Guild shrub passerines	-0.14	113	-0.28	0.78
**Grazing**	**0.02**	**11**	**2.37**	**0.04**
Guild dabbling ducks × Dredging	0.004	113	0.17	0.87
Guild diving omnivores × Dredging	0.01	113	0.49	0.63
**Guild piscivore × Dredging**	**-0.05**	**113**	**-2.07**	**0.04**
Guild swans × Dredging	-0.01	113	-0.49	0.63
Guild geese × Dredging	0.02	113	0.70	0.49
Guild waders × Dredging	0.02	113	1.01	0.31
**Guild b-h gull × Dredging**	**0.06**	**113**	**2.39**	**0.02**
**Guild rallids and bittern × Dredging**	**0.07**	**113**	**3.15**	**0.002**
Guild open hab. passerines × Dredging	0.01	113	0.57	0.57
Guild shrub passerines × Dredging	0.04	113	1.49	0.14

Supplementary Table S6. Summary table of the management actions in the final LMM explaining the abundance of staging migrant birds on the studied wetlands during their autumn migration.

Independent variable	Parameter estimate (b)	df	t	P
**Birds prior to management**	**0.005**	**310**	**5.53**	**<.0001**
**Water area**	**0.009**	**310**	**4.22**	**<.0001**
Land area	0.003	310	1.16	0.25
Guild diving omnivores	-0.45	310	-1.80	0.07
**Guild diving piscivores**	**-1.22**	**310**	**-4.81**	**<.0001**
**Guild swans**	**-1.76**	**310**	**-6.97**	**<.0001**
**Guild geese**	**-1.24**	**310**	**-4.94**	**<.0001**
**Guild waders**	**-2.23**	**310**	**-8.81**	**<.0001**
**Guild b-h gull**	**-2.36**	**310**	**-9.32**	**<.0001**
**Guild dabbling ducks × Grazing**	**0.04**	**310**	**3.20**	**0.002**
Guild diving omnivores × Grazing	0.02	310	1.76	0.08
Guild diving piscivores × Grazing	-0.004	310	-0.33	0.74
Guild swans × Grazing	0.02	310	1.35	0.18
**Guild geese × Grazing**	**0.06**	**310**	**4.31**	**<.0001**
**Guild waders × Grazing**	**0.09**	**310**	**6.52**	**<.0001**
**Guild b-h gull × Grazing**	**0.03**	**310**	**2.06**	**0.04**

Supplementary Table S7. Summary table of the final LMM where the abundance of staging migrant birds on the studied wetlands during their spring migration was explained by the total costs of wetland management.

Independent variable	Parameter estimate (b)	df	t	P
**Birds prior to management**	**0.005**	**331**	**7.88**	**<.0001**
**Water area**	**0.007**	**331**	**3.58**	**0.0004**
Land area	0.002	331	0.63	0.53
Guild diving omnivores	0.23	331	0.98	0.33
Guild diving piscivores	0.04	331	0.15	0.88
**Guild swans**	**-1.36**	**331**	**-5.78**	**<.0001**
**Guild geese**	**-1.75**	**331**	**-7.45**	**<.0001**
**Guild waders**	**-1.53**	**331**	**-6.53**	**<.0001**
**Guild b-h gull**	**-1.07**	**331**	**-4.59**	**<.0001**
**Guild dabbling ducks × Cost**	**0.00001**	**331**	**3.60**	**0.0004**
Guild diving omnivores × Cost	-0.0000001	331	-0.02	0.98
Guild diving piscivores × Cost	0.0000009	331	0.26	0.79
Guild swans × Cost	0.000003	331	0.95	0.34
**Guild geese × Cost**	**0.000009**	**331**	**2.68**	**0.008**
**Guild waders × Cost**	**0.00002**	**331**	**6.92**	**<.0001**
**Guild b-h gull × Cost**	**0.00001**	**331**	**3.94**	**0.0001**

Supplementary Table S8. Summary table of the final LMM where the abundance of breeding birds on the studied wetlands was explained by the total costs of wetland management.

Independent variable	Parameter estimate (b)	df	t	P
**Birds prior to management**	**0.006**	**113**	**4.48**	**<.0001**
**Water area**	**0.003**	**12**	**4.16**	**0.001**
Land area	-0.0003	12	-0.18	0.86
Guild diving omnivores	-0.06	113	-0.11	0.91
Guild diving piscivores	0.68	113	1.23	0.22
**Guild swans**	**-1.76**	**113**	**-3.19**	**0.002**
**Guild geese**	**-2.83**	**113**	**-5.12**	**<.0001**
Guild waders	-0.84	113	-1.52	0.13
**Guild b-h gull**	**-3.32**	**113**	**-6.02**	**<.0001**
**Guild rallids**	**-2.63**	**113**	**-4.75**	**<.0001**
**Guild open hab. passerines**	**-1.46**	**113**	**-2.65**	**0.009**
Guild shrub passerines	0.08	113	0.14	0.89
Guild dabbling ducks × Cost	0.000002	113	0.48	0.63
Guild diving omnivores × Cost	0.000001	113	0.01	0.99
**Guild diving piscivores × Cost**	**-0.00001**	**113**	**-2.25**	**0.03**
Guild swans × Cost	-0.000004	113	-0.99	0.32
Guild geese × Cost	0.000001	113	-0.08	0.94
Guild waders × Cost	0.000005	113	1.07	0.29
**Guild b-h gull × Cost**	**0.00002**	**113**	**5.10**	**<.0001**
**Guild rallids × Cost**	**0.00001**	**113**	**2.63**	**0.01**
**Guild open hab. passerines × Cost**	**0.00001**	**113**	**2.51**	**0.01**
Guild shrub passerines × Cost	0.000003	113	0.63	0.53

Supplementary Table S9. Summary table of the final LMM where the abundance of staging migrant birds on the studied wetlands during their autumn migration was explained by the total costs of wetland management.

Independent variable	Parameter estimate (b)	df	t	P
**Birds prior to management**	**0.005**	**310**	**6.30**	**<.0001**
**Water area**	**0.008**	**310**	**3.95**	**0.0001**
Land area	0.005	310	1.67	0.1
Guild diving omnivores	-0.26	310	-0.98	0.33
**Guild diving piscivores**	**-1.05**	**310**	**-3.96**	**0.0001**
**Guild swans**	**-1.62**	**310**	**-6.08**	**<.0001**
**Guild geese**	**-1.25**	**310**	**-4.75**	**<.0001**
**Guild waders**	**-2.28**	**310**	**-8.59**	**<.0001**
**Guild b-h gull**	**-2.35**	**310**	**-8.81**	**<.0001**
**Guild dabbling ducks × Cost**	**0.00001**	**310**	**2.94**	**0.004**
Guild diving omnivores × Cost	0.000001	310	0.35	0.73
Guild diving piscivores × Cost	-0.000004	310	-1.06	0.29
Guild swans × Cost	0.000002	310	0.47	0.64
**Guild geese × Cost**	**0.00002**	**310**	**4.04**	**0.0001**
**Guild waders × Cost**	**0.00002**	**310**	**6.28**	**<.0001**
**Guild b-h gull × Cost**	**0.000008**	**310**	**2.13**	**0.03**

Supplementary Table S10. Summary table of the management actions in the final LMM explaining the abundance of EU red-listed staging spring migrant birds.

Independent variable	Parameter estimate (b)	df	t	P
**Birds prior to management**	**0.004**	**31**	**2.12**	**0.04**
**Water area**	**0.01**	**31**	**3.78**	**0.0007**
Land area	0.003	31	0.61	0.55
**Grazing**	**0.06**	**31**	**3.85**	**0.0006**

Supplementary Table S11. Summary table of the management actions in the final LMM explaining the abundance of staging autumn migrant birds belonging to EU Birds Directive Annex I.

Independent variable	Parameter estimate (b)	df	t	P
**Birds prior to management**	**0.04**	**31**	**3.71**	**0.0008**
Water area	0.002	31	0.89	0.38
Land area	0.003	31	0.92	0.36
**Grazing**	**0.03**	**31**	**2.48**	**0.02**

Supplementary Table S12. Summary table of the management actions in the final LMM explaining the abundance of EU red-listed staging autumn migrant birds.

Independent variable	Parameter estimate (b)	df	t	P
**Birds prior to management**	**0.02**	**26**	**5.02**	**<.0001**
Water area	0.005	26	1.69	0.10
Land area	-0.002	26	-0.45	0.65
**Grazing**	**0.03**	**26**	**2.32**	**0.03**
**Cutting/harrowing**	**0.03**	**26**	**2.15**	**0.04**
**Dredging**	**0.04**	**26**	**2.08**	**0.048**

Supplementary Table S13. Summary table of the final LMM where the abundance of EU red-listed staging autumn migrant birds was explained by the total costs of wetland management.

Independent variable	Parameter estimate (b)	df	t	P
**Birds prior to management**	**0.02**	**28**	**5.43**	**<.0001**
Water area	0.003	28	1.13	0.27
Land area	0.001	28	0.28	0.78
**Cost**	**0.000008**	**28**	**2.27**	**0.03**

Supplementary Table S14. Summary table of the non-significant variables removed from the model explaining the abundance of staging migrant birds on the studied wetlands during their spring migration.

Independent variable	df	F	P
Guild × Tree removal	6, 310	1.39	0.22
Tree removal	1, 316	0.48	0.49
Guild × Grazing	6, 317	1.70	0.12

Supplementary Table S15. Summary table of the non-significant variables removed from the model explaining the abundance of breeding birds on the studied wetlands.

Independent variable	df	F	P
Guild × Tree removal	9, 87	0.22	0.99
Tree removal	1, 9	1.89	0.20
Guild × Cutting/harrowing	9, 96	0.89	0.53
Cutting/harrowing	1, 8	0.53	0.49
Guild × Grazing	9, 106	1.77	0.08
Grazing	1, 10	2.85	0.12

Supplementary Table S16. Summary table of the non-significant variables removed from the model explaining the abundance of staging migrant birds on the studied wetlands during their autumn migration.

Independent variable	df	F	P
Guild × Tree removal	6, 289	0.87	0.52
Tree removal	1, 295	0.16	0.69
Guild × Dredging	6, 296	1.29	0.26
Dredging	1, 302	0.03	0.86
Guild × Cutting/harrowing	6, 304	0.56	0.76
Cutting/harrowing	1, 310	2.32	0.13

Supplementary Table S17. Summary table of the non-significant variables removed from the model explaining the abundance of EU red-listed staging spring migrants on the studied wetlands.

Independent variable	df	F	P
Tree removal	1, 30	2.65	0.11
Dredging	1, 29	0.65	0.43
Cutting/harrowing	1, 28	0.29	0.59

Supplementary Table S18. Summary table of the non-significant variables removed from the model explaining the abundance of the birds belonging to EU Birds Directive Annex I species staging during spring migration.

Independent variable	df	F	P
Cutting/harrowing	1, 28	0.45	0.51
Dredging	1, 29	0.31	0.58
Tree removal	1, 30	3.26	0.08

Supplementary Table S19. Summary table of the non-significant variables removed from the model explaining the abundance of EU red-listed staging autumn migrants on the studied wetlands.

Independent variable	df	F	P
Tree removal	1, 25	0.0008	0.98

Supplementary Table S20. Summary table of the non-significant variables removed from the model explaining the abundance of the birds belonging to EU Birds Directive Annex I species staging during autumn migration.

Independent variable	df	F	P
Tree removal	1, 25	1.83	0.19
Dredging	1, 26	0.15	0.70
Cutting/harrowing	1, 27	0.37	0.55
Grazing	1, 28	3.43	0.07
